# Research on Non-Pooling YOLOv5 Based Algorithm for the Recognition of Randomly Distributed Multiple Types of Parts

**DOI:** 10.3390/s22239335

**Published:** 2022-11-30

**Authors:** Zehua Yu, Ling Zhang, Xingyu Gao, Yang Huang, Xiaoke Liu

**Affiliations:** 1 Guangxi’s Key Laboratory of Manufacturing Systems and Advanced Manufacturing Technology, Guilin University of Electronic Technology, Guilin 541004, China; 2 National Space Science Center, Chinese Academy of Sciences, Beijing 100190, China; 3 University of Chinese Academy of Sciences, Beijing 100049, China

**Keywords:** parts classification, YOLOv5, non-pooling

## Abstract

Part cleaning is very important for the assembly of precision machinery. After cleaning, the parts are randomly distributed in the collection area, which makes it difficult for a robot to collect them. Common robots can only collect parts located in relatively fixed positions, and it is difficult to adapt these robots to collect at randomly distributed positions. Therefore, a rapid part classification method based on a non-pooling YOLOv5 network for the recognition of randomly distributed multiple types of parts is proposed in this paper; this method classifies parts from their two-dimensional images obtained using industrial cameras. We compared the traditional and non-pooling YOLOv5 networks under different activation functions. Experimental results showed that the non-pooling YOLOv5 network improved part recognition precision by 8% and part recall rate by 3% within 100 epochs of training, which helped improve the part classification efficiency. The experiment showed that the non-pooling YOLOv5 network exhibited improved classification of industrial parts compared to the traditional YOLOv5 network.

## 1. Introduction

Manufacturing, storage, and transport processes often leave surface contaminants, such as grease and fine foreign matter, on the parts of a mechanical device, which need to be cleaned before assembly. The effective cleaning of these parts using a mechanical cleaner can help avoid such contaminants from compromising the service life and use quality of the mechanical device. Often, multiple types of cleaned parts are scattered randomly in the collection area after the cleaning process is completed, and these parts need to be stacked neatly based on predefined rules. Traditional robots cannot be programmed to collect parts that are randomly distributed; they can only collect parts from predefined positions. Furthermore, vision-guided robots cannot perform tasks that involve collecting multiple types of parts because of their inability to conduct part classification. The current solution to this problem is manual collection or cleaning of only one type of part at a time; however, this greatly increases the production cost. Therefore, a non-pooling YOLOv5 network is proposed in this paper for the recognition of multiple randomly distributed types of parts to improve collection efficiency and minimize production costs.

Thus far, several research studies have focused on approaches to realize part collection. For example, Tekin et al. [1] proposed a convolutional neural network (CNN) that can perform both object classification and object pose computation. The CNN used three-dimensional (3D) boundary boxes and was similar to the You Only Look Once (YOLO) algorithm in the training process; however, the method required the computation of complex 3D information and therefore suffered from insufficient recognition speed. Peng et al. [2] proposed a pixel-wise voting network (PVNet) that first classified objects in images using a CNN, and then computed the object pose information. PVNet obtained object poses using only two-dimensional (2D) images, but this had to be preceded by object classification using another network. Furthermore, PVNet did not have an object detection function, and it was not suitable for mechanical parts with complex shapes and small volumes. Iriondo et al. [3] proposed a deep-learning-based solution for the collection of randomly distributed parts that classified parts from high-precision 2D and 3D images by analyzing the material, shape, color, and texture of the parts using deep learning. However, the solution had a low computational speed and was unsuitable for industrial production. Qi et al. [4] proposed a deep-learning model capable of direct recognition of point clouds; this model segmented an integral point cloud into a maximum of four parts and was trained on all parts of the integral point clouds in the training model. The model recognized objects by analyzing the shape and parts contained in their integral point clouds; however, this model only considered large objects and was therefore unsuitable for the recognition of small objects. The main problem is that the current detection accuracy of randomly distributed parts is not enough.

The object classification problem is similar to the object detection problem. Early object detection algorithms include sliding-window methods, wherein a fixed-size detection window is used to detect fixed-size objects pixel-by-pixel based on the predefined stride. However, these methods suffer from low detection speeds and poor efficiency. In 2012, CNN rose to prominence with the emergence of AlexNet [5], and it was used for object detection. Region-based CNN (R-CNN) [6], which was the first attempt to use CNN for object detection, was proposed in 2014. R-CNN detected objects by pre-selecting candidate regions in the input image instead of scanning all regions in the image, as in the sliding-window methods, and this greatly improved the detection efficiency. Fast RCNN [7] and Faster RCNN [8] were developed based on R-CNN, and they showed better performance. Furthermore, Simonyan and Zisserman proposed a visual geometry group (VGG) network [9] in 2015. The VGG network was the first attempt to use small kernels for convolutions, and it had a uniform kernel size of 3 × 3 for all convolutional layers. In the same year, Redmond et al. proposed the epoch-making YOLO algorithm [10].

One of the major challenges in object detection is the detection of multi-scale objects associated with whether the detector can accept multi-scale images as input. Ma et al. [11] used a lightweight feature fusion single-shot multibox detector (L-SSD) for garbage classification; this was incorporated with feature pyramid networks (FPNs) to solve the multi-scale object detection problem. Zhao et al. [12] incorporated a modified FPN into a lightweight VGG network to solve the multi-scale object detection problem. The FPN used the inherent multi-scale pyramidal hierarchical architecture of deep convolutional networks to construct feature pyramids, and it was a laterally connected top-down structure. Zhang et al. proposed an FPN that combined top-down and bottom-up structures [13]. 

For the multi-scale image input problem, one deficiency of the R-CNN [6] was its inability to process multi-scale input images. This problem was solved using a spatial pyramid pooling net (SPP-Net). Mathew and Kurian used an SPP-Net model to identify malicious code variants that caused computer crash screens [14]. Wang et al. used the Tiny YOLOv3 for vehicle detection and used an SPP-Net to increase the number of feature channels, which helped improve the feature extraction capacity of the network [15]. Thus, the incorporation of SPP can help solve the problem that R-CNN cannot process multi-scale input images; however, SPP layers are not capable of backpropagation. 

The performance of a neural network is affected by the selection of the activation function. Sigmoid and tanh functions were used as activation functions when neural networks had just emerged. The activation function used by Jarrett [16] was the absolute value of a tanh function; furthermore, that used in the earliest image recognition network, AlexNet, was rectified linear units (ReLU) [5]. The activation function used in traditional YOLOv5 networks is sigmoid linear units (SiLU), which is a special case of the Swish activation function discovered by Ramachandran et al. [17] using automatic search techniques. The Mish activation function was proposed by Misra [18] in 2019 and was used in YOLOv4.

Inspired by human vision works, some deep learning networks have begun to add attention mechanisms to improve the networks. The attention mechanism can be applied to machine translation, data analysis, image recognition, etc. He [19] reviewed the attention mechanism in the field of machine translation. He classified attention mechanisms into soft attention mechanisms and hard attention mechanisms. The soft attention mechanism is the average information of the input, while the hard attention mechanism is the maximum information of the input. Cai [20] improved the online advertising click-through rate (CTR) prediction using the attention mechanism. Du [21] proposed a target detector based on a gradient harmonized mechanism (GHM) and an attention mechanism to realize synthetic aperture radar (SAR) target detection in complex scenes. Gao [22] proposed a method of food image recognition in an oven based on a mixed attention mechanism. Woo [23] proposed the Convolutional Block Attention Module (CBAM).

This study aims to solve the problem of collecting multiple randomly distributed types of parts for industrial production. The problems of the recognition of multi-scale input images and the selection of the activation function are discussed and analysed for the YOLOv5 network; a non-pooling YOLOv5 network is developed. The differences in performance between the traditional YOLOv5 network, the non-pooling YOLOv5 network, and the YOLOv5 with Convolutional Block Attention Module (CBAM) are illustrated via performance experiments, and experimental conclusions are provided.

The remainder of this article is organized as follows. Section 2 investigates the related work on YOLOv5, the multi-scale image input problem, and CBAM. Section 3 explicitly describes the improvement of YOLOv5 with a modified structure called spatial pyramid convolutions (SPC). Section 4 presents and analyses the experimental results, which show that the YOLOv5 network with an SPC structure using the Mish activation function improved compared to the traditional YOLOv5 network. Section 5 is the conclusion of this article.

## 2. Related Work

### 2.1. Traditional YOLOv5 Network

The input image is divided into *S × S* equal-sized cells, with each cell containing *B* detection boxes; the detection boxes for each cell have the same initial size. During network training, YOLOv5 scans every cell and detects where the annotated center of an object’s true box is located among all *S × S* cells. If the center is located in cell *S_ij_*, then the *B* detection boxes in cell *S_ij_* are used to determine the predicted box of the object. The presence of the target object in the cell of each box is verified; if the target object is present in the cell, the object is classified. A tensor with a size of *S × S* × (*B* × 5 + *C*) is obtained by aggregating the information about the presence and classification of the object; here, *C* represents the number of classes to which the target object possibly belongs. *Pr*(*Class_i_*|*ob_j_*) denotes the probability that the target object belongs to the *i*-th class. Each detection box has five parameters: horizontal coordinate *x* and vertical coordinate *y* of the center, width *w* and height *h* of the detection box, and the confidence score of the detection box. The confidence score is calculated as:

(1) $$Pr\left(obj\right)*IOU_{truth}^{pred}$$

where $Pr\left(obj\right)$ represents the probability that the target object is present in the detection box, with $Pr\left(obj\right)$ = 1 if the target object is present in the detection box and $Pr\left(obj\right)$ = 0 otherwise. $IOU_{truth}^{pred}$ represents the intersection-over-union (*IOU*) of the detection box and the true box.

The per class confidence score of each detection box (*Score*) is defined as the product of the probability that the target object belongs to the *i*-th class $Pr\left(Class_{i}|obj\right)$ and the confidence score of the detection box $Pr\left(obj\right)*IOU_{truth}^{pred}$; it is expressed as

(2) $$Score=Pr\left({Class}_{i}|obj\right)*Pr\left(obj\right)*IOU_{truth}^{pred}$$

A threshold is defined once the per class confidence of each detection box is obtained; then, a non-maximum suppression is used to remove the detection boxes with a confidence lower than the threshold. The remaining detection boxes, referred to as predicted boxes, are presented as the final detection results.

**Figure 1:**
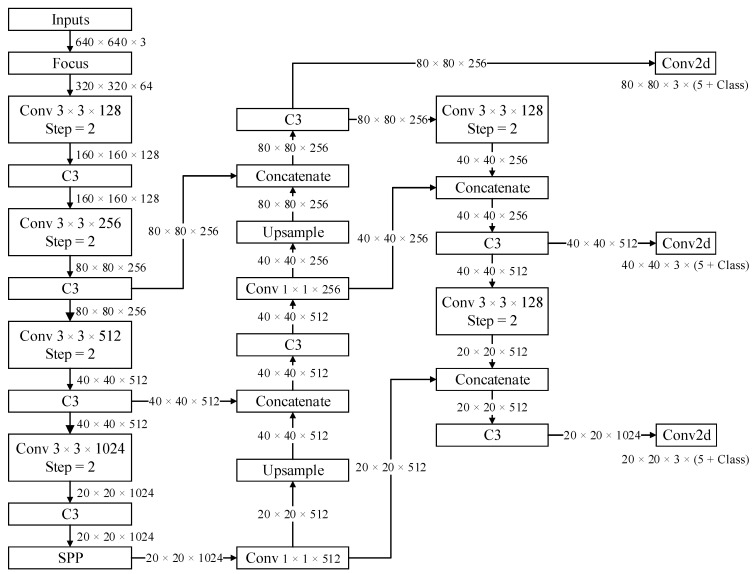
Traditional YOLOv5 network.

Figure 1 shows the traditional YOLOv5 network. In this network, five rounds of downsampling (convolutions with a step of 2) are performed on the image, and this results in the loss of some image information. In YOLOv5, a C3 structure is used to solve the problem of image information loss. A C3 structure is a simplified content security policy (CSP) structure, and this is an improvement of the residual structure. The path aggregation network (PAN) module, which is an improvement of the FPN module, is used in the neck layer of YOLOv5. The FPN module integrates high-level semantic information into low-level semantic information, whereas the PAN proposed by Liu et al. [24] integrates low-level semantic information into high-level semantic information based on FPN.

### 2.2. Training on Multiscale Images

**Figure 2:**
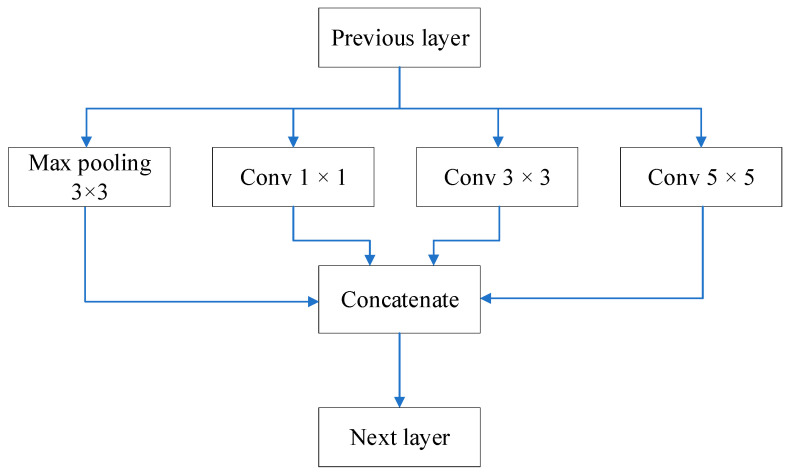
Basic Inception Architecture.

GoogLeNet [25] created a new architecture, referred to as the Inception architecture. Inception uses dense matrices to approximate optimal local sparse structures. This architecture only changes the depth of the tensors and not the width and height, as indicated in Figure 2.

The convolution applied to an input tensor $X_{in}\in R_{W_{i}\times H_{i}\times C_{i}}$ using step *s*, padding *p*, kernel size *N* × *N,* and outputs $X_{out}\in R_{W_{o}\times H_{o}\times C_{o}}$ is expressed as:

(3) $$X_{out}=CovN_{C_{o}}^{s,p}\left(X_{in}\right)$$

The maximum pooling applied to an input *X_in_* using step *s*, padding *p*, kernel size *N* × *N*, and outputs *X_out_* is expressed as:

(4) $$X_{out}=MaxPoolN^{s,p}\left(X_{in}\right)$$

For *n* tensors (*X*_1_, *X*_2_, …, *X_n_*) with width *W*, height *H*, and depths *C*_1_, *C*_2_, …, *C_n_*, respectively, the output from the concatenation of these *n* tensors in the depth direction $X_{out}\in R_{W\times H\times C_{o}}$ can be expressed as:

(5) $$X_{out}=Cat\left(X_{1},X_{2},\ldots X_{n}\right)$$

where $C_{o}=\overset{n}{\underset{i=1}{\sum }}C_{i}$ designates the input tensor of the Inception architecture as $X_{in}\in R_{W\times H\times C_{i}}$, and then, the basic Inception architecture can be expressed as:

(6) $$X_{out}=Cat\left(MaxPool3^{1,1}\left(X_{in}\right),Cov1_{C_{1}}^{1,0}\left(X_{in}\right),Cov3_{C_{2}}^{1,1}\left(X_{in}\right),\;Cov5_{C_{3}}^{1,2}\left(X_{in}\right)\right)$$

where *C*_1_, *C*_2_, and *C*_3_ represent the depths of the output tensors from the layers and $X_{out}\in R_{W\times H\times C_{o}}$.

Inception architecture contains convolutional kernels of different sizes corresponding to different receptive fields. The Inception architecture adds a 1 × 1 convolutional layer before 3 × 3 and 5 × 5 convolutional layers to avoid the excessively large computational load in a large kernel (for example, a 5 × 5 kernel); this reduces the dimensions of these large kernels.

(7) $$X_{1}=Cov1_{C_{1}}^{1,0}\left(X_{in}\right)$$

where $X_{in}\in R_{W\times H\times C_{i}}$ represents the input tensor, and $X_{1}\in R_{W\times H\times C_{1}}$ represents the output tensor from the first convolutional layer.

This is followed by the convolution of *X_C_*_1_ using a step and padding of 1, and a kernel size of 3 × 3.

(8) $$X_{21}=Cov3_{C_{21}}^{1,1}\left(X_{1}\right)$$

and the convolution using a step of 1, padding of 2, and kernel size of 5 × 5.

(9) $$X_{22}=Cov5_{C_{22}}^{1,2}\left(X_{1}\right)$$

where $X_{1}\in R_{W\times H\times C_{1}}$ and $X_{22}\in R_{W\times H\times C_{22}}$.

This dimensionality reduction operation contributes to reduced computational load; the reduction in computational load is related to the number of 1 × 1 convolutional kernels in that layer, the number of image input channels, and the ratio of the number of 1 × 1 convolutional kernels in that layer to the number of image input channels.

In the dimensionality-reducing Inception architecture, the maximum pooling of *X_in_* is followed by a 1 × 1 convolutional layer, which is given as

(10) $$X_{23}=Cov1_{C_{1}}^{1,0}\left(MaxPool3^{1,1}\left(X_{in}\right)\right)$$

where $X_{23}\in R_{W\times H\times C_{1}}$.

*X*_21_, *X*_22_, and *X*_23_ are concatenated in the depth direction as

(11) $$X_{out}=Cat\left(X_{21},\;X_{22},X_{23}\right)$$

where $X_{out}\in R_{W\times H\times C_{o}}$.

**Figure 3:**
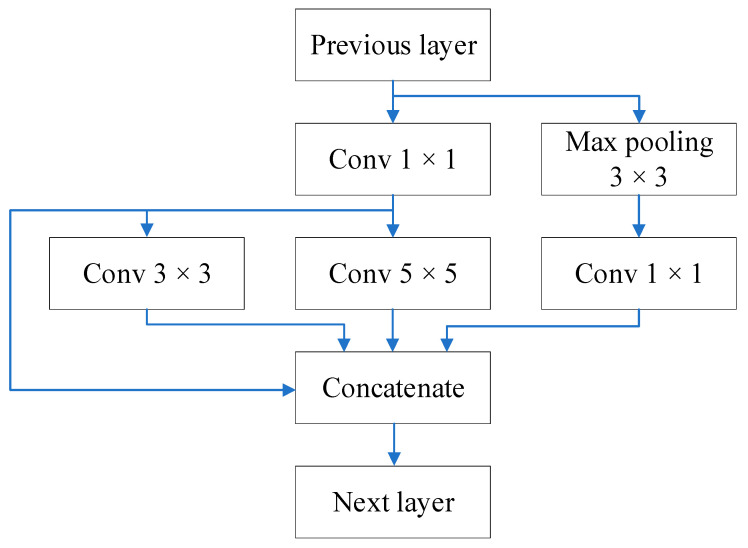
Dimensionality-reducing inception architecture.

Figure 3 shows the dimensionality-reducing Inception architecture.

Common CNNs can accept only images of a fixed size as the input. The input images that do not meet the predefined size are scaled prior to the network training. However, this approach is not only time-consuming but also leads to unsatisfactory training results caused by the deformations and warps applied to the input images. The SPP-Net architecture [26] was developed to solve this problem. This architecture is similar to the Inception architecture in that the tensors are first fed into a 1 × 1 convolutional layer for dimensionality reduction prior to the next-step operations; finally, the resulting tensors are concatenated in the third dimension and transmitted to the next layer. The difference in both architectures is that the convolutions are performed in Inception, whereas maximum pooling is performed in SPP.

**Figure 4:**
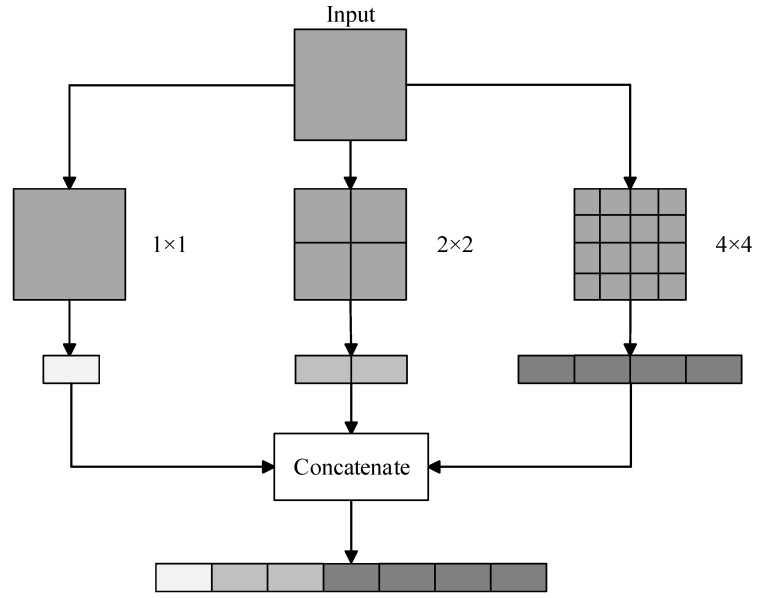
Spatial pyramid pooling (SPP) operation.

Figure 4 shows an illustration of the SPP operation, where the input image is segmented at three scales. The input image is divided into 4 × 4 = 16 cells, which are subjected to maximum pooling to obtain a four-dimension tensor. Then, the input image is divided into 2 × 2 = 4 cells, which are subjected to maximum pooling to obtain a two-dimension tensor. Finally, the input image is divided into 1 × 1 = 1 cell, or the entire image is treated as a single cell and subjected to maximum pooling; this yields a one-dimension tensor. These tensors are concatenated in the depth direction, which helps to obtain the output tensor.

**Figure 5:**
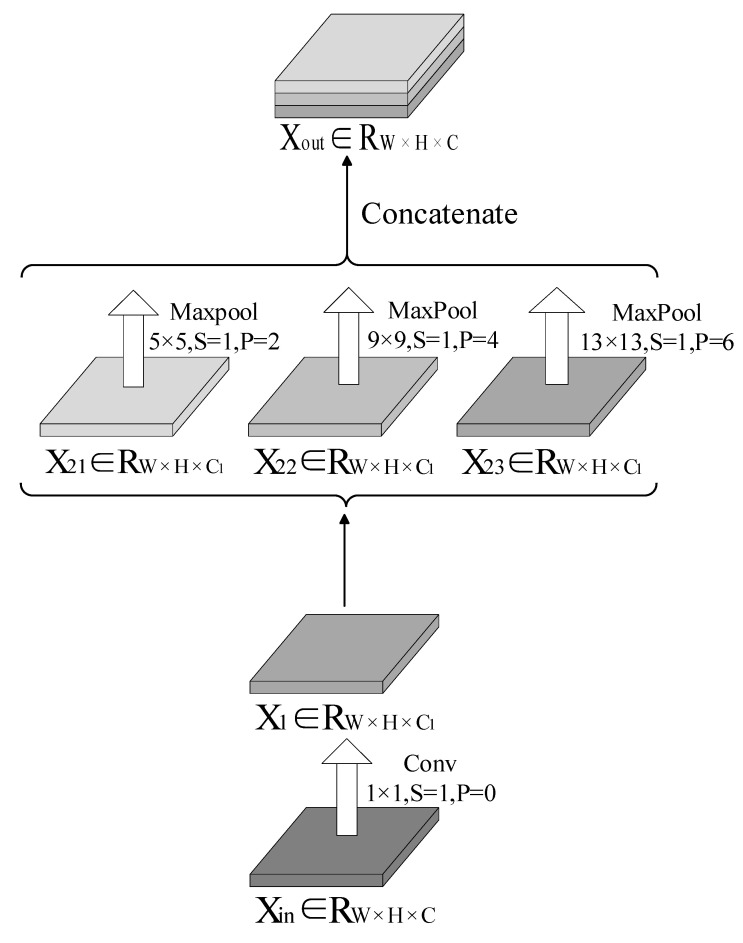
SPP structure.

YOLOv5 is inspired by this idea and is incorporated with the SPP module, as shown in Figure 5. The output tensor *X_out_* from the SPP structure of YOLOv5 is given as

(12) $$X_{out}=Cat\left(MaxPool5^{1,2}\left(X_{1}\right),\;MaxPool9^{1,4}\left(X_{1}\right),MaxPool13^{1,6}\left(X_{1}\right)\right)$$

where

(13) $$X_{1}=Cov1_{C_{1}}^{1,0}\left(X_{in}\right)$$

where $X_{1}\in R_{W\times H\times C_{1}}$, $X_{in}\in R_{W\times H\times C_{i}}$, and $X_{out}\in R_{W\times H\times C}$.

## 3. Proposed Method

The algorithm proposed in this study was designed to recognize images of randomly distributed parts obtained using industrial cameras. The images were transmitted to a master computer, where the class of each part was analyzed and determined using the non-pooling (Non-Pooling, NP) YOLOv5. Finally, the classification results were transmitted to a robot that placed the parts into designated areas based on their classification.

The performance of the proposed NP-YOLOv5 network was tested using two different activation functions (SiLU and Mish). Comparative tests were performed to demonstrate the superior performance of the NP-YOLOv5 network over the traditional YOLOv5 network and the effects of different activation functions on the networks. The performance of the traditional YOLOv5 network using different activation functions was also tested.

### 3.1. Non-Pooling YOLOv5

The convolutional network obtained the minimum weight and bias gradient by calculating the loss function, which helped ensure the convergence and accuracy of the network. Unlike the convolutional layers, the pooling layers do not consider weight and bias. Therefore, the propagation of the error term is realized by upsampling the error term during backpropagation through the pooling layers. This characteristic of the pooling layers allows them to have the advantage of rapid computational speed; however, it results in significant information loss. During backpropagation in single-scale maximum pooling, only the maxima during forward propagation are recovered. All other values are assigned a value of 0. In contrast, backpropagation cannot compute the derivative of the tensor input into the SPP layer from the derivative of the output tensor in multi-scale maximum pooling (such as the SPP structure) because the output from the backpropagation at one scale overwrites the output from that at another scale. Therefore, multi-scale maximum pooling structures like the SPP cannot perform backpropagation. This problem can be solved by replacing the pooling layer with a convolutional layer. A new spatial pyramid convolution (SPC) structure was proposed based on this idea. With the SPC structure incorporated into YOLOv5, the entire YOLOv5 network has a non-pooling layer, and it consists exclusively of convolutional layers.

**Figure 6:**
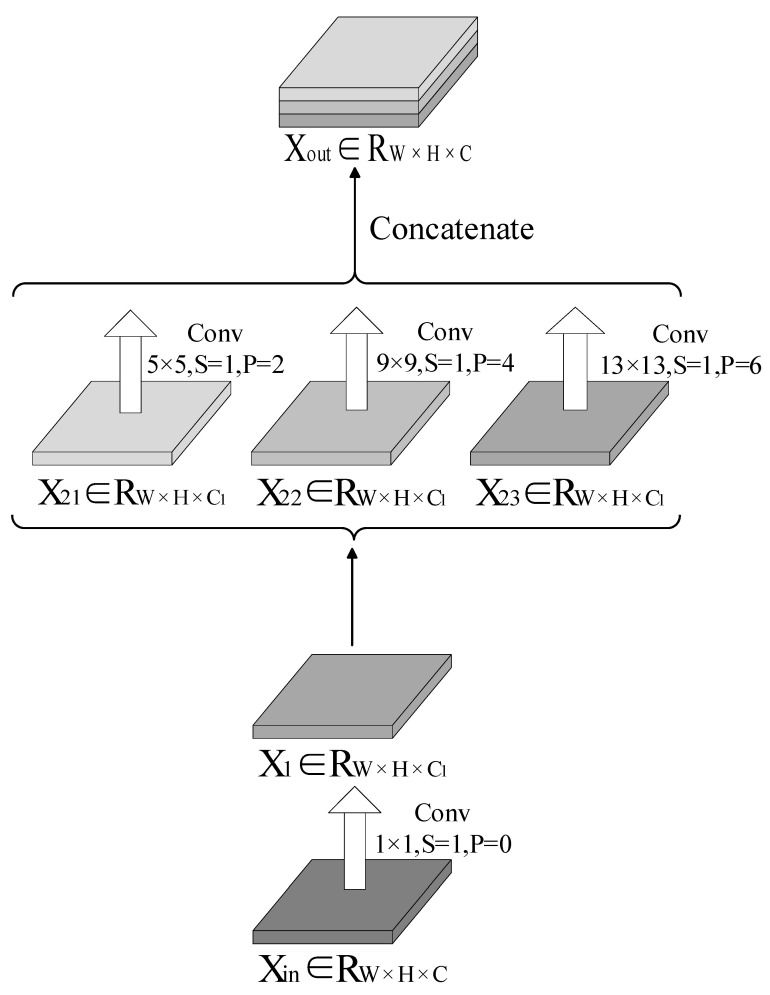
Spatial pyramid convolutions (SPC) structure.

Figure 6 shows an illustration of the SPC structure. In the SPC structure, there are *C_1_* convolutions in each convolutional layer. The input tensor $X_{in}\in R_{W\times H\times C}$, where $C_{1}=\frac{C}{4}$. Furthermore, *X_in_* is convolved in the first layer using a step of 1, padding of 0, and kernel size of 1 × 1:

(14) $$X_{1}=Cov1_{C_{1}}^{1,0}\left(X_{in}\right)$$

where $X_{1}\in R_{W\times H\times C_{1}}$.

This is followed by the convolutions of *X*_1_ first using a step of 1, padding of 2, and kernel size of 5 × 5 as

(15) $$X_{21}=Cov5_{C_{1}}^{1,2}\left(X_{1}\right)$$

Then, using a step of 1, padding of 4, and kernel size of 9 × 9,

(16) $$X_{22}=Cov9_{C_{1}}^{1,4}\left(X_{1}\right)$$

Finally, using a step of 1, padding of 6, and kernel size of 13 × 13,

(17) $$X_{23}=Cov13_{C_{1}}^{1,6}\left(X_{1}\right)$$

where $X_{21},\;\;X_{22},\;\mathrm{and}\;X_{23}\in R_{W\times H\times C_{1}}$.

Furthermore, *X*_21_, *X*_22_, and *X*_23_ are concatenated in the third dimension, which yields the output tensor as

(18) $$X_{out}=Cat\left(X_{21},\;X_{22},X_{23}\right)$$

where $X_{out}\in R_{W\times H\times C}$.

### 3.2. Selection of Activation Function

Both sigmoid and tanh functions are saturating activation functions and have a value range of [0, 1]; however, the use of a saturating function leads to a vanishing gradient problem during training. ReLU has a value range of (0, +∞) and is a non-saturating function that can effectively avoid the vanishing gradient problem during training. The ReLU activation function selects the maximum value between x and 0, and it is expressed as:

(19) $$f\left(x\right)=\max\left(0,\;x\right)$$

The Swish activation function has a value range of (–0.28, +∞) and is a non-saturating function. Swish is smoother than ReLU, which is non-differentiable at *x* = 0, and it is also more conducive to gradient computation and updating. Swish is expressed as:

(20) $$f\left(x\right)=\frac{x}{1+e^{-\beta x}}$$

**Figure 7:**
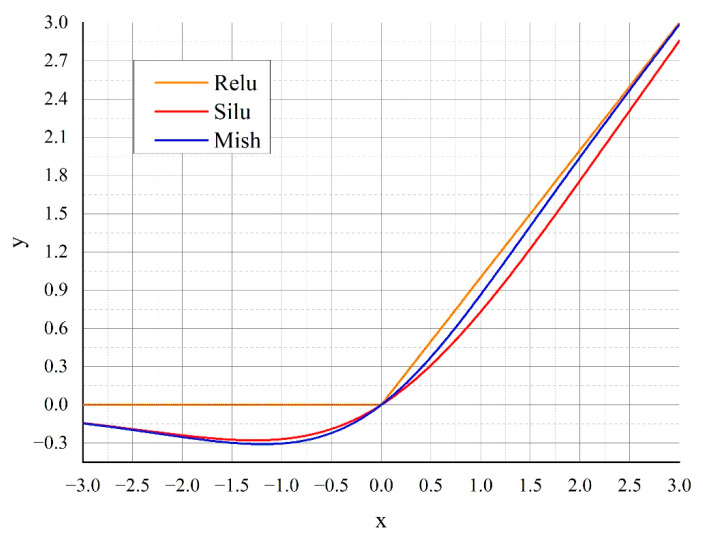
Comparison of activation functions.

Swish is superior to ReLU because it is non-monotonic when *x* < 0, as indicated in Figure 7. This region is controlled by parameter *β* of Swish; when *β* = 1, the effect is the best.

(21) $$f\left(x\right)=\frac{x}{1+e^{-x}}$$

SiLU is used in the traditional YOLOv5 network.

The Mish activation function has a value range of (–0.31, +∞) and is a non-saturating function. Mish is approximate to Swish, as indicated in Figure 7. Despite Mish being approximate to Swish, it can more easily eliminate the problem of the big jumps of loss function, i.e., the dying ReLU problem [18], during training. Thus, Mish is more conducive to gradient computation and updating than Swish. Furthermore, the accuracy of Mish does not decrease significantly with an increase in network size. Moreover, Mish is highly noise resistant, and its accuracy is minimally affected by initialization. Therefore, Mish was selected as the activation function for the proposed algorithm.

Mish is expressed as:

(22) $$f\left(x\right)=x\cdot tanh\left(ln\left(1+e^{x}\right)\right)$$

where

(23) $$tanh\left(x\right)=\frac{e^{2x}-1}{e^{2x}+1}$$

## 4. Experiments and Results

### 4.1. Evaluation Indices of Detection Performance

The mean average precision (*mAP*) and loss function were used as performance indices in this study. The *mAP* of a model is related to its precision, recall, and number of classes (*n_c_*), and it is expressed as:

(24) $$mAP=\frac{1}{n_{c}}\int_{0}^{1}P\left(R\right)dR$$

Precision is defined as the ratio of the number of true positives (positives in the ground truth correctly predicted as positives), *TP*, to the total number of predicted positives; it is expressed as

(25) $$P=\frac{TP}{TP+FP}$$

where *FP* represents the number of false positives (negatives in the ground truth that are incorrectly predicted as positives).

Recall represents the ratio of *TP* to the number of positives in the ground truth in the predicted sample; it is expressed as

(26) $$R=\frac{TP}{TP+FN}$$

where *FN* represents the number of false negatives (positives in the ground truth that are incorrectly predicted as negatives) in the predicted set.

The prediction outcome (positive or negative) is evaluated based on the degree of overlap measured using *IOU* between the predicted and true boxes. The prediction outcome is positive if the *IOU* is larger than the predefined threshold (set to 0.5 in this study); otherwise, it is negative.

The loss function of YOLOv5 is:

(27) $$loss=l_{loc}+l_{obj}+l_{cls}$$

where *l_loc_* represents the localization loss of positive predictions defined as complete IOU (*CIOU*) loss; *l_obj_* represents the *CIOU* loss of the detection and true boxes defined as binary cross entropy (BCE) loss; and *l_cls_* represents the classification loss of positive predictions defined as BCE loss.

The three losses are expressed as

(28) lloc=1−CIOUlobj=−∑ntCIoUilnc^i+1−CIoUiln1−c^iNlcls=−∑i∈pos∑j∈classCijlnC^ij+1−Cijln1−C^ijnt

where *CIOU*, c^i, *nt*, *N*, *C_ij_* ∈ [0, 1], and C^ij represent the *CIOU* of the detection and true boxes [27], confidence of the predicted box, number of positive predictions, total number of positive and negative predictions, whether the *i*-th detection box contains the *j*-th class object, and prediction output, i.e., predicted probability, respectively.

Compared with *CIOU*, *IOU* cannot compute loss and has slow convergence and low localization accuracy when the detection and true boxes do not intersect. The *CIOU* of detection and true boxes are expressed as

(29) CIOU=IOUtruthpred−ρ2b,b^c2+αν

where

(30) ρ2b,b^=xb−xb^2−yb−yb^2α=ν1−IOUtruthpred+νν=4π2arctanwh−arctanw^h^2

where $IOU_{truth}^{pred}$, *b*, b^, ρ2b,b^, *c*, *w* and *h*, and w^ and h^ represent the *IOU* of the detection and true boxes, coordinates of the center of the true box (*x_b_*, *y_b_*), coordinates of the center of the true box xb^, yb^, Euclidean distance between *b* and b^, length of the diagonal of the minimum bounding rectangle of the true and predicted boxes, width and height of the true box, respectively, and width and height of the predicted boxes, respectively.

### 4.2. Comparison of Different Algorithms

**Figure 8:**
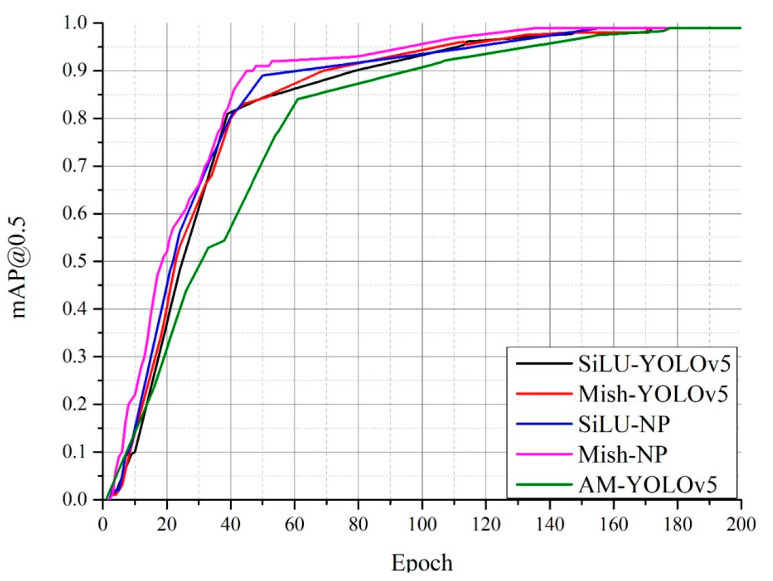
Comparison of the mAPs of the algorithms at *IOU* = 0.5.

Figure 8 shows a comparison of the mAPs of the NP-YOLO network equipped with the Mish activation function (Mish-NP network) and the other networks at *IOU* = 0.5. The Mish-NP network had a mAP above 0.9 after 45 epochs of training and outperformed the three other networks. The NP-YOLO network equipped with the SiLU activation function (SiLU-NP network) had a mAP above 0.8 after 40 epochs of training and a mAP of 0.95 after 110 epochs of training. The mAPs of the traditional YOLOv5 (SiLU-YOLOv5) network equipped with the Mish activation function (Mish-YOLOv5 network) and the SiLU-YOLOv5 network reached 0.8 after 40 epochs of training. The Mish-YOLOv5 network took 20 less epochs of training to increase the mAP from 0.8 to 0.95 than the SiLU-YOLOv5 network. The traditional YOLOv5 network equipped with the CBAM attention mechanism (AM-YOLOv5) had a mAP above 0.8 after 55 epochs of training and needed another 100 epochs to have a mAP above 0.95.

**Figure 9:**
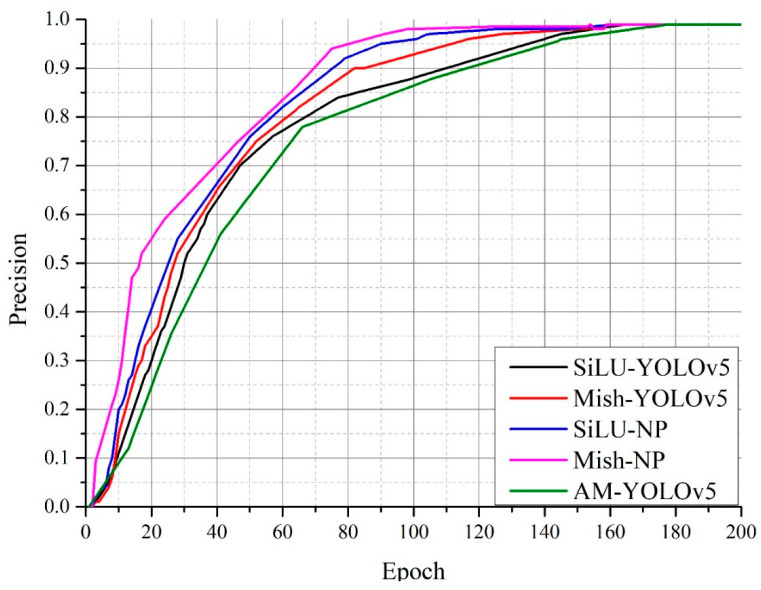
Comparison of the precisions of algorithms.

Figure 9 shows a comparison of the precision of the Mish-NP network and the other networks. The Mish-NP network had a precision above 0.95 after 80 epochs of training, thereby outperforming the other three networks. The SiLU-NP, Mish-YOLOv5, and SiLU-YOLOv5 networks required 90, 115, and 140 epochs of training, respectively, to increase precision above 0.95. The AM-YOLOv5 network required 5 more epochs than the SiLU-YOLOv5 network. The Mish-YOLOv5 network had better precision than the SiLU-YOLOv5 network; however, it had poorer precision than the Mish-NP network.

**Figure 10:**
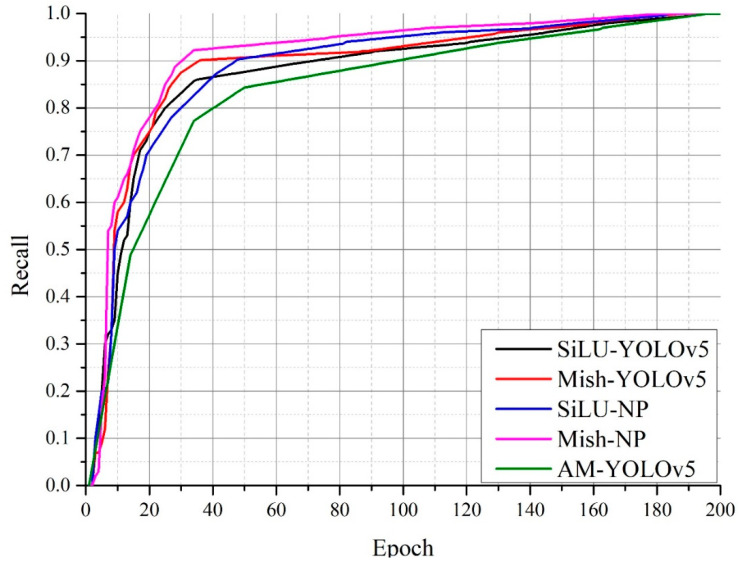
Comparison of the recalls of the algorithms.

Figure 10 shows a comparison of the recall rates of the Mish-NP network and the other three networks. The recall rate of the Mish-NP network reached 0.9 after 30 epochs of training and 0.95 after 80 epochs of training, thus outperforming the other three networks. The recall rate of the SiLU-NP network increased slowly compared to that of the other three networks; however, it reached 0.95 after 95 epochs of training, which was faster than that of the SiLU-YOLOv5 and Mish-YOLOv5 networks. The recall rate of the SiLU-YOLOv5 networks did not reach above 0.9 until after 70 epochs of training, and it reached above 0.95 after 130 epochs of training, which significantly underperformed against the Mish-NP network. Mish-YOLOv5 showed a better recall rate compared to the SiLU-YOLOv5 network, and it was the closest to the Mish-NP network. The recall rate of the Mish-YOLOv5 network reached 0.9 after only 35 epochs of training; however, it reached above 0.95 very slowly. It reached 0.95 until 125 epochs. The recall rate of the AM-YOLOv5 reached above 0.9 after 95 epochs, and at the same epochs, the recall rate of the Mish-NP reached above 0.97.

**Figure 11:**
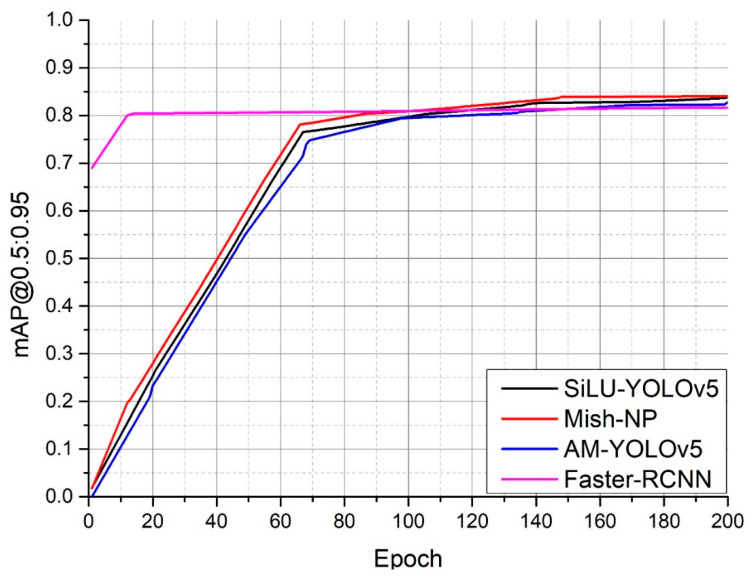
Comparison of the average of mAPs from *IOU* = 0.5 to *IOU* = 0.95 for algorithms.

Figure 11 shows the average mAP of the SiLU-YOLOv5, Mish-NP, AM-YOLOv5, and Faster-RCNN networks when *IOU* = 0.5:0.05:0.95. Although the convergence rate of the Faster-RCNN network is faster than that of the SiLU-YOLOv5 network, the convergence rate of mAP@0.5:0.95 of Faster-RCNN is only around 0.81, while the mAP@0.5:0.95 of the AM-YOLOv5 can reach 0.828, the mAP@0.5:0.95 of the SiLU-YOLOv5 network can reach 0.837 and the mAP@0.5:0.95 of the Mish-NP can reach 0.84.

Table 1 presents the performance results for the four methods after 100 epochs of training. Compared with the SiLU-YOLOv5 networks, the Mish-NP networks showed significantly improved performance. The precision improved by a relatively large margin, from 0.88 to 0.9, whereas the recall rate and mAP at *IOU* = 0.5 improved by a relatively small margin, from 0.93 to 0.96.

**Figure 12:**
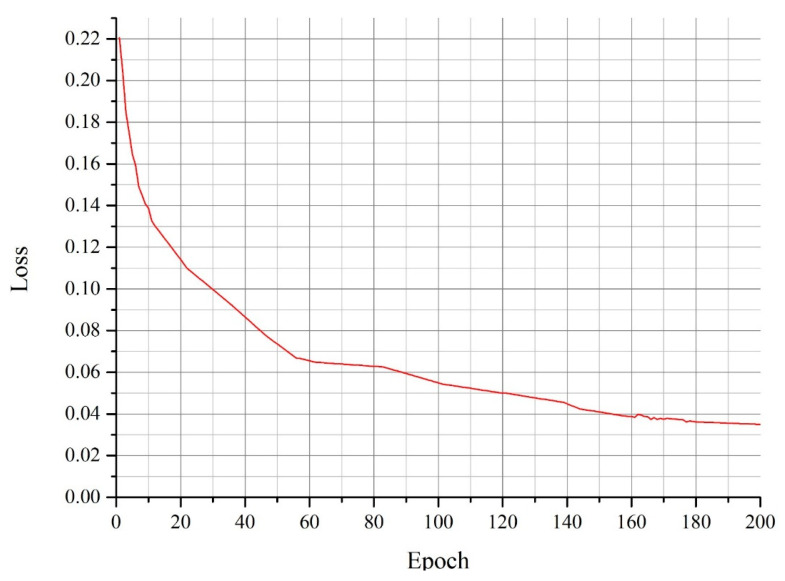
Loss of Mish-NP.

The rate of convergence of loss represents the rate of convergence of the network. The faster the loss decreases, the faster the network converges. Figure 12 shows the rate of convergence of the loss of the Mish-NP network. The loss decreased quickly in the first 60 epochs of training. From the 60th epoch, the rate of decrease slowed down markedly, which indicates that the model gradually matured. From the 140th epoch, the loss stabilized, which indicates that the model matured and did not undergo further marked changes.

**Figure 13:**
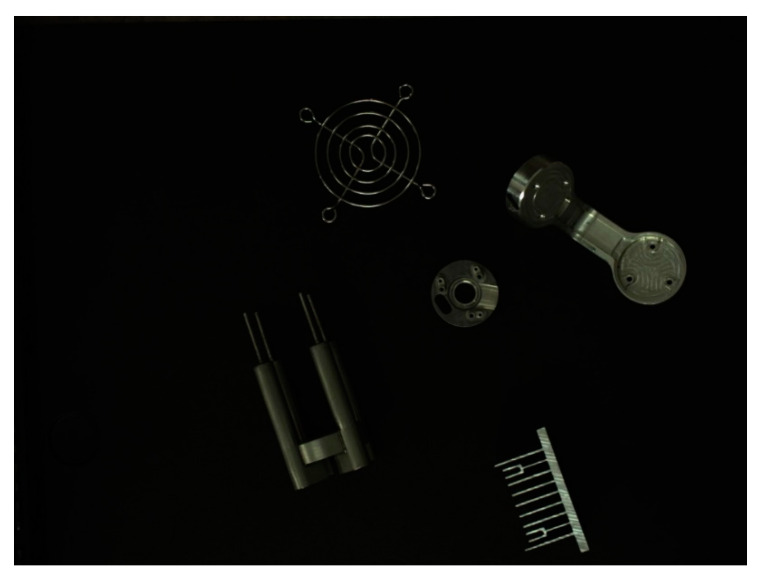
Example of an input image.

**Figure 14:**
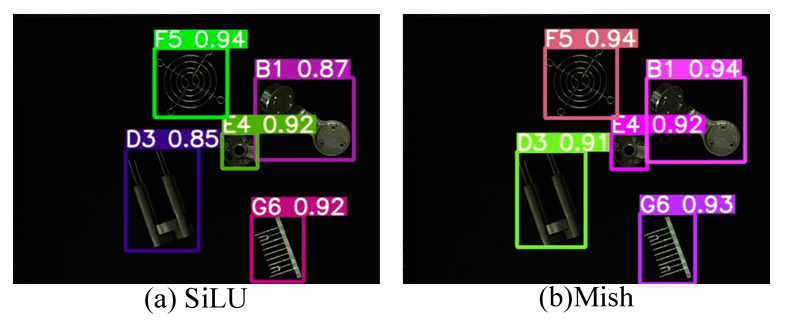
Results of Traditional YOLOv5.

**Figure 15:**
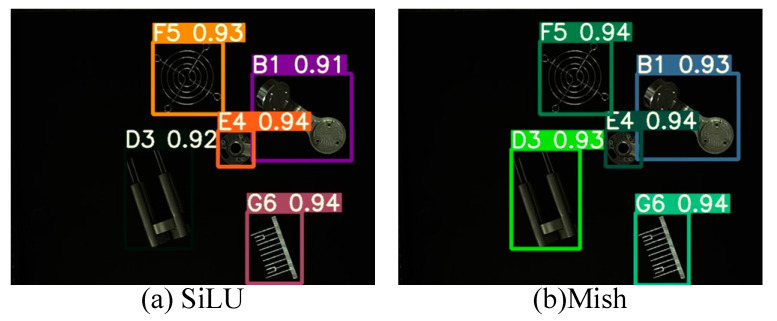
Results of NP-YOLOv5.

**Figure 16:**
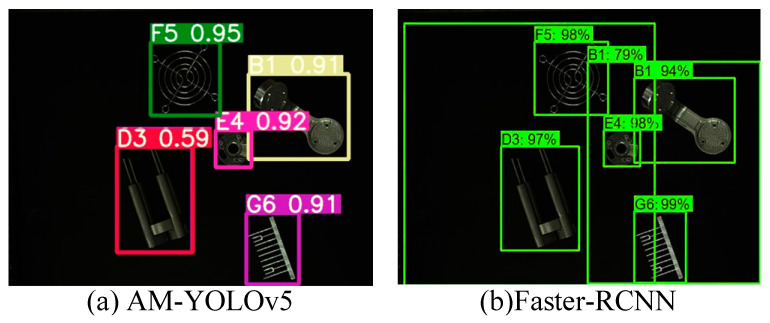
Results of AM-YOLOv5 and Faster-RCNN.

Figure 13 shows the images of the target objects of detection. Figure 14a,b present the object recognition results yielded by the SiLU-YOLOv5 and Mish-YOLOv5 networks, respectively; Figure 15a,b show the recognition results yielded by the SiLU-NP and Mish-NP networks, respectively; Figure 16a,b show the recognition results yielded by the AM-YOLOv5 and Faster-RCNN networks, respectively. The decimals or percentages in these figures indicate the confidence scores of the predicted boxes yielded by the networks. A comparison of Figure 14a,b and Figure 15a,b indicates that the Mish activation function yielded higher part recognition confidence scores than the SiLU activation function after the same number of epochs of training; this is especially true for the D3-type part, which indicates that Mish contributed to easier part classification after the same number of epochs of training.

A comparison of Figure 14a and Figure 15a, Figure 14b and Figure 15b shows that the NP-YOLOv5 networks achieved a higher part-recognition confidence than the SiLU-YOLOv5 networks after the same number of epochs of training, especially for the D3-type part. This indicates that the NP-YOLOv5 network outperformed the in-part classification after the same number of epochs of training.

A comparison of Figure 14a, Figure 15b, and Figure 16a shows that the AM-YOLOv5 network is not better enough than the Mish-NP network and is sometimes not better enough than the SiLU-YOLOv5 in our datasets. A comparison of Figure 14a, Figure 15b, and Figure 16a shows that the Faster-RCNN network is not better enough than the SiLU-YOLOv5 in our datasets.

### 4.3. Experimental Result

The Mish activation function not only outperformed the SiLU activation function on the SiLU-YOLOv5 network but also performed well on the NP-YOLO network; this indicates that Mish outperformed SiLU. Therefore, the Mish activation function was suitable for the NP-YOLO network. Figure 8, Figure 9 and Figure 10 indicate that the SiLU-YOLOv5 network equipped with the better-performing Mish activation function still underperformed against the NP-YOLO network equipped with the SiLU activation function. Furthermore, a comparison of Figure 14b and Figure 15a showed a small gap in the actual performance, but a comparison of Figure 14a and Figure 15b showed that the Mish-YOLOv5 network had markedly improved performance when compared with the SiLU-YOLOv5 network, and it achieved the best performance among the four networks. Thus, the NP-YOLO network equipped with the Mish activation function showed the best performance among the four networks. The traditional YOLOv5 network equipped with the CBAM attention mechanism and Faster-RCNN network is not better than the traditional YOLOv5 network in our datasets.

## 5. Conclusions

A new non-pooling YOLOv5-based algorithm was proposed for the recognition of multiple types of parts. The algorithm was designed to solve the problem of classifying multiple types of parts in the collection area after they are cleaned and of placing them in the designated areas according to the classification. The SPC structure was proposed to replace the SPP structure in the traditional YOLOv5 network. The effects of different activation functions on the different structures were also discussed.

The experimental results showed that YOLOv5 equipped with the SPC structure (NP network) outperformed the original YOLOv5 and had higher recall and precision rates for the same activation function. The Mish activation function was more conducive to part classification than the SiLU activation function, and it contributed to higher recall and precision rates under the same network structure. Thus, the NP-YOLO network equipped with the Mish function proposed in this study outperformed the traditional YOLOv5 network (SiLU-YOLOv5) in part classification. The non-pooling YOLOv5 algorithm has a certain improvement in parts detection. Furthermore, the experimental results confirmed that the Mish-equipped NP-YOLO system had higher precision in part classification and higher efficiency in the collection of multiple types of parts after they were cleaned in a mixed manner.

The focus of this research is the effect of the convolutional layer replacement pooling layer in the traditional YOLOv5 network. We have not discussed whether the other networks have the same effect. Meanwhile, the datasets we used are self-created datasets of parts. In the future, we will consider whether it is suitable to improve more networks using this method and whether it is suitable for more datasets.

## Figures and Tables

**Table 1:** Comparison of four networks.

Method	mAP@0.5	Precision	Recall
YOLOv5	0.93	0.88	0.93
Mish-YOLOv5	0.94	0.94	0.93
SiLU-NP	0.94	0.95	0.95
Mish-NP	0.96	0.96	0.96
AM-YOLOv5	0.91	0.86	0.90
Faster-RCNN	0.85	0.82	0.86

## Data Availability

Data underlying the results used in this paper are not publicly available at this time but may be obtained from the authors upon reasonable request.
